# Minocycline and Risperidone Prevent Microglia Activation and Rescue Behavioral Deficits Induced by Neonatal Intrahippocampal Injection of Lipopolysaccharide in Rats

**DOI:** 10.1371/journal.pone.0093966

**Published:** 2014-04-04

**Authors:** Furong Zhu, Yingjun Zheng, Yu-qiang Ding, Yong Liu, Xianghui Zhang, Renrong Wu, Xiaofeng Guo, Jingping Zhao

**Affiliations:** 1 Mental Health Institute of The Second Xiangya Hospital, National Technology Institute of Psychiatry, Key Laboratory of Psychiatry and Mental Health of Hunan Province, Central South University, Changsha, Hunan, China; 2 Department of General Psychiatry, Guangzhou Brain Hospital, Affiliated Hospital of Guangzhou Medical College, Guangzhou, Guangdong, China; 3 Department of Anatomy and Neurobiology, Tongji University School of Medicine, Shanghai, China; University of Insubria, Italy

## Abstract

**Background:**

Various signs of activation of microglia have been reported in schizophrenia, and it is hypothesized that microglia activation is closely associated with the neuropathology of schizophrenia.

**Methods:**

Neonatal intrahippocampal injection of lipopolysaccharide (LPS), an activator of microglia, was performed in rats at postnatal day 7 (P7), and they were separately given saline, risperidone (0.5 mg/kg), minocycline (40 mg/kg) or a combination of both of them at P42 for consecutive 14 days. Behavioral changes (locomotion activity, social interaction, novel object recognition and prepulse inhibition) were examined and the number of microglia was assessed by using immunohistochemistry in adulthood.

**Results:**

The adult rats in LPS-injected group showed obvious behavioral alteration (e. g. deficits in social interaction, novel object recognition and prepulse inhibition) and a dramatic increase of number of activated microglial cells in the hippocampus and other brain regions such as cerebral cortex and thalamus compared to those in saline-injected group. Interestingly, application of either minocycline, risperidone or both of them significantly rescued behavioral deficits and attenuated microglia activation.

**Conclusion:**

Our results suggest that inhibition of microglia activation may be one of mechanisms underlying the antipsychotic effect of minocycline and risperidone.

## Introduction

Schizophrenia is a chronic and debilitating illness which affects about 1% of the world population. The onset of full-blown schizophrenia is typically in late adolescence or in early adulthood, and the distinct symptoms in schizophrenia are commonly referred to as positive, negative and cognitive symptoms [1]. The etiology of schizophrenia remains unclear, while there has been a growing amount of evidence suggesting that the neuroinflammation and immunogenetics may be involved [2]. Microglia are the primary reservoirs of pro-inflammatory cytokines such as interleukin-6 (IL-6), tumor necrosis factor-α (TNF-α) and interferon-γ (IFN-γ) and act as antigen-presenting cells in the brain [3]. They function as a key cellular element and the major players in innate immunity in the central nervous system (CNS) [4]. In many aspects, the neuropathology of schizophrenia has recently been suggested to be closely associated with microglia activation. For example, a postmortem study [5] found activated microglia in schizophrenic brain particularly in the frontotemporal regions, and a positron emission tomography (PET) study [6], [7] reported an increased number of microglial cells in patients with schizophrenia.

Lipopolysaccharide (LPS) is a protein-free endotoxin derived from the cell wall of gram-negative bacteria following multiplication or lysis, and it causes the release of a variety of pro-inflammatory mediators from immune cells [8]. LPS is a representative ligand for Toll-like receptor 4 (TLR4), and TLR4 signaling has an important role in microglial activation and thus is widely used as a common activator of microglia [9]. Feleder et al [10] found that neonatal intrahippocampal injection of LPS induced prepulse inhibition (PPI) deficits and a persistent elevation of the cytokines in several brain regions. We thus supposed that neonatal intrahippocampal LPS-induced schizophrenia-like behaviors may be related with microglia activation, and prevented by antibiotics and/or antipsychotics.

It has been reported the second-generation tetracycline minocycline produces neuroprotective effects in some animal models of schizophrenia [11], [12]. In this study, we aimed to assess whether minocycline and risperidone are able to rescue the behavioral deficits and prevent microglia activation induced by injecting LPS into the ventral hippocampus (VH) in rats.

## Methods and Materials

### Animals

**Figure 1:**
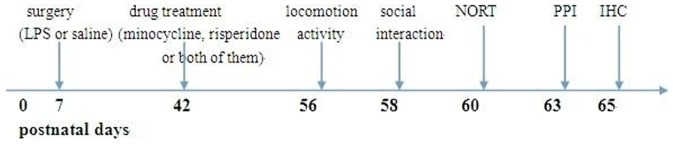
The timeline of treatment, behavioral testing and IHC.

Timed pregnant Sprague-Dawley female animals were obtained at embryonic day 17 to 19 from Slac Jingda Laboratory Animal Co., Ltd. (Changsha, Hunan, China) and individually housed with free access to food and water in a temperature and humidity controlled environment with a 12-hour light/12-hour dark cycle (lights on at 7:00 AM). All animal experiments in the present study were approved by the Animal Care and Use Committee of Central South University, Changsha, China. The timeline of treatment, behavioral testing and IHC was shown in Figure 1.

### Neonatal Ventral Hippocampal LPS Injection

The surgery procedure was carried out as described previously [10]. The pups were left undisturbed until P7, and female pups and underdeveloped male pups were euthanized. At P7, healthy male pups (15–20 g) received a bilateral injection of either saline or LPS (Serotype 055:B5; Sigma, St. Louis, MO, USA) in the VH. Pups were anesthetized by hypothermia, secured to a platform placed in a stereotaxic apparatus (David Kopf; Tujunga, CA, USA), and the scalp incised. Rats received bilateral infusions (0.3 μl per side at 0.15 μl/min) of LPS (10 μg/μl) dissolved in 0.9% sterile saline into the VH at 3 mm caudal to Bregma, 3.5 mm lateral to midline, and 5 mm from the surface of the skull. Control rats received bilateral infusions of 0.9% sterile saline. The infusion needle was left in place for 3 min after each infusion. The wound was closed, and pups were placed on a warming pad until their respiration and locomotor activity returned to normal. Pups were returned to their home cages, and were weaned at approximately P23. After weaning, the rats were housed in group (4 rats in one cage).

### Drug treatment

At P42, these rats received either neonatal VH-injection saline or LPS were treated with saline, minocycline, risperidone and both of them, respectively. And thus in this study there were eight groups: saline+saline, saline+minocycline, saline+ risperidone, saline+minocycline+risperidone, LPS+saline, LPS+minocycline, LPS+risperidone, and LPS+minocycline+risperidone. Minocycline hydrochloride (Wyeth, China) was freshly dissolved in saline, and risperidone (Janssen, China) was freshly dissolved in 0.1 M tartaric acid (1 mg risperidone in 7.5 μl) first and then diluted with saline. Minocycline (40 mg/kg) [11] or risperidone (0.5 mg/kg) [13] were intragastric administrated once a day in a volume of 10 ml/kg for consecutive 14 days. Behavioral examinations were performed on the next day (P56) after the last injection, which corresponds to late adolescence/young adult stage [14].

### Behavioral assessment tools

#### Locomotor activity

We first used Open-field (ENV-515; Med Associates Inc., St. Albans, VT, USA) to assess locomotor activity (n = 8 for each group). It was monitored in a 43.2×43.2×30.5 acrylic box for 10 min. During the test, the box was cleaned with 75% ethanol to avoid any possible instinctive odorant cues, and this cleaning was also performed in the following behavioral tests. Locomotion was detected by the sensors and transmitted to a computer for statistical analysis.

#### Social interaction test

The adult rats were tested in the Open field apparatus after 30 min acclimatization. Two individually housed rats in the same group were randomly selected and placed in the box. Social interaction includes the following behaviors: following or approaching the test partner, mounting or crawling over the test partner, sniffing or grooming any part of the body of the test partner [15].Their behaviors were recorded by a video camera which was placed above the arena for 10 min, and the number of social contacts and total time were measured by stopwatch by the person who was blind to the experiment design.

#### Novel object recognition test

The novel object recognition test (NORT) (n = 8 for each group) was conducted to assess non-spatial memory in a 43.2×43.2×30.5 box. A preceding 10 min habituation was done to reduce the contribution of anxiety and stress on the outcome. The animals were videotaped in both training and retention sessions. During the training session, two novel objects were symmetrically fixed to the floor of the box with a distance of 10 cm from the walls. Each animal was allowed to explore in the box for 10 min. An animal was considered to be exploring the object when its head was facing the object (the distance between the head and object is an approximately 1 cm or less) or it was touching or sniffing the object [16]. After the training, the rats were immediately returned to their home cages, and the box and objects were cleaned with 75% ethanol to avoid any possible instinctive odorant cues. Retention tests were carried out on the next day of the training. During the retention test, each rat was placed back into the same box, with one of the objects used during training replaced by a novel object. Each rat was then allowed to freely explore for 10 min, and the time spent exploring each object was recorded by the person who was blind to the experiment design. Exploratory preference, the ratio of the amount of time spent exploring any one of the two objects (training session) or the novel object (retention test session) over the total time spent exploring both objects, was used to measure the memory performance.

#### Prepulse inhibition (PPI)

We performed PPI test several days after the novel object recognition test. Rats were individually placed in a sound attenuated startle chamber (San Diego Instruments, San Diego, CA, USA) with a 70 decibel (dB) background white noise. After a 5-min period of adaptation, the PPI test was initiated with pseudorandom trials every 20 to 30 sec. Either pulse (120 dB), prepulse (75 dB, 80 dB, or 85 dB), no pulse, or prepulsepulse were delivered. Trials lasted for 23 min and 8–10 repetitions of pulse or prepulse+pulse trials were acquired, while null or prepulse only trials were repeated 5 times for each prepulse amplitude.PPI was calculated as the percent inhibition of the startle amplitude evoked by the pulse alone: % PPI = 100 (magnitude on pulse alone trial - magnitude on prepulse+pulse trial/magnitude on pulse alone trial). Following behavioral testing, the rats were subjected to immunohistochemistry.

### Immunohistochemistry

Rats which have been subjected to all behavioral tests mentioned above were deeply anesthetized and transcardially perfused with 0.01 M phosphate buffer solution (PBS, pH 7.4) followed by 4% paraformaldehyde (PFA) in PBS. Brains were removed, post-fixed in 4% PFA for 4–6 h, cyoprotected with 20% sucrose in PBS overnight, embedded with opti-mum cutting temperature compound (OCT), cut into 12 μm-thick sections using a freezing microtome (CM1950; Leica, Germany) mounted onto superfrost plus glass slides, and stored at −70°C for immunohistochemistry. Four sets of serial sections were obtained from each brain, and one of them was used for immunostaining. Three sections were analyzed per rat.

Immunohistochemistry was performed as described previously [17]. Sections were re-hydrated in PBS, heated in 0.1 M citric acid buffer (pH 6.0) for 3×5 min in a microwave and allowed to cool in the buffer for 30 min to retrieve the antigen. After being washed in PBS, endogenous peroxidase was blocked by incubation in 0.3% hydrogen peroxide in 50% methanol in distilled water for 30 min. Sections were washed in PBS and pre-incubated in a blocking solution containing 6% bovine serum albumin (BSA) and 0.03% Triton X-100 in PBS for 60 min at room temperature. Then, sections were incubated in a humidified chamber with the rabbit primary antibody against ionized calcium binding adapter molecule 1(Iba1) (1∶400; Wako, Japan) in 3% BSA in PBS overnight at 4°C. After several washes in PBS, sections were incubated with biotinylated goat anti-rabbit secondary antibody (1∶300; CWBIO, China) for 60 min at room temperature, followed by incubation with the avidin-biotin complex (Vectastain ABC Kit; Vector Laboratories, Burlingame, CA, USA). Immunoreactive signals were detected by using 3,3′-diaminobenzidinetetrahydro-chloride (Boster, China) as a chromogen.

Images were captured using a digital camera (Olympus DP72) attached to an Olympus microscope (Olympus BX51) and Cellsens standard 1.6 computer program. For statistical analysis, two sections between Bregma −4.8 mm and −5.6 mm were randomly selected; at this rostrocaudal extent, the whole VH is present in one section. Three images were taken from the upper, middle and lower portions of VH and one image respectively from the ventrobasal thalamus(Th) and cerebral cortex (Cx, which refers to the neocortex) were taken on both sides using x10 objective. Iba-1 positive cells on each side of VH, Th and Cx were counted from the images, pooled together and the averaged cell number was used for statistical comparison. Cells were counted by a research assistant who was blind to experimental design.

### Statistical analysis

The data were expressed as the mean ± standard error of the mean (S.E.M.). All analyses were performed using the Statistical Package for the Social Sciences (SPSS 19.0) software. Differences in microglial cell number and behavioral data (locomotor activity, social interaction and novel object recognition test) in experimental groups were first determined by the two-way Analysis of Variance (ANOVA), if the interaction effect was statistically significant, the one-way ANOVA was followed by Bonferroni post hoc test. PPI experiments were analyzed using repeated measures two-way ANOVA with group treatment as a between-subjects factor and prepulse intensity as a within-subjects factor. When appropriate, group means at individual dB levels were compared by one-way ANOVA, followed by Bonferroni post hoc test. P values were considered significant when P<0.05.

## Results

### Behavioral tests

#### Locomotor activity

**Figure 2:**
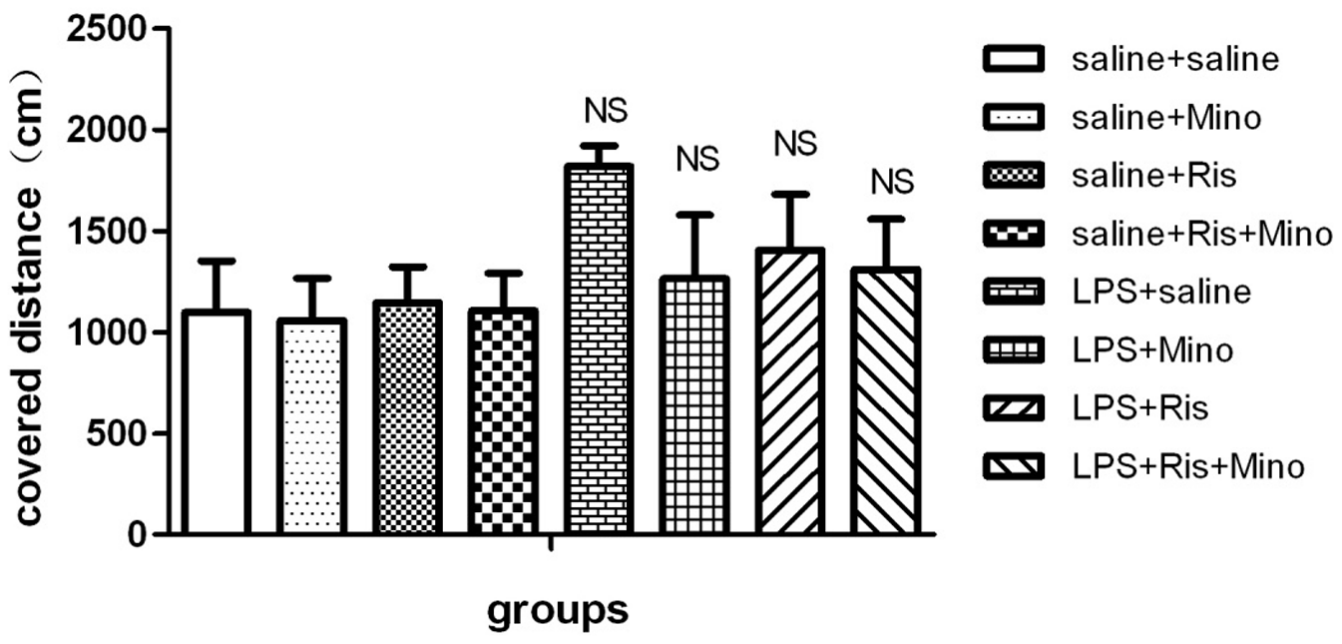
Effects of minocycline, risperidone and combination of these two supplementation and LPS treatment on open-field task. NS represents no significance. The data is shown as means ± SEM. n = 8 for each group.

As shown in Figure 2, ANOVA analysis revealed no significant effect among the eight groups [F(7,56) = 1.193, P>0.5]. The result revealed that neonatal intrahippocampal injection of LPS resulted in an increase of locomotor activity in rats compared with those received the intrahippocampal injection of saline and minocycline was able to reduce the increased locomotion,but it did not reach the level of statistical significance.

#### Social interaction test

**Figure 3:**
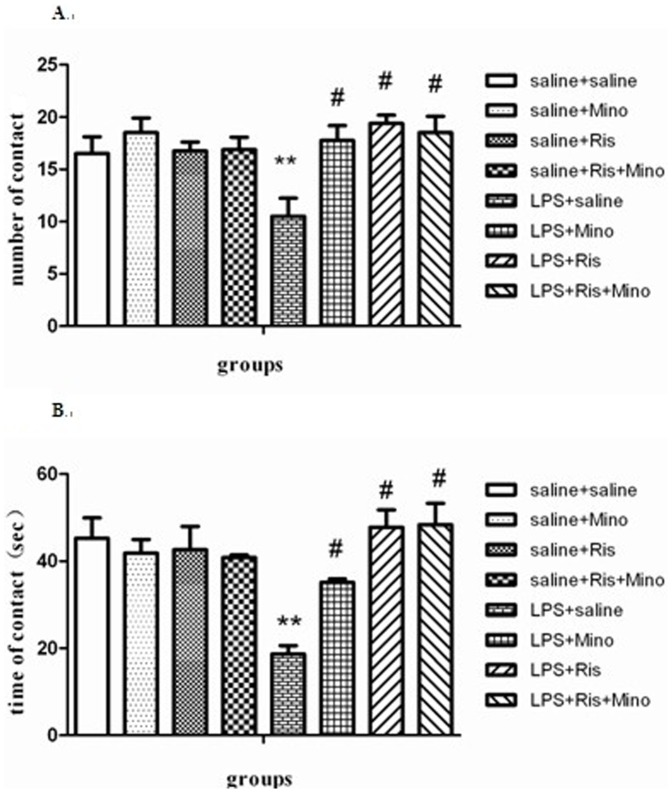
Effects of minocycline, risperidone and combination of these two on LPS-induced social interaction behaviors shown by number of contact (Figure 3A) and time spent in contact (Figure 3B). The data are shown as means ± SEM. n  =  8 for each group. ** P<0.01, compared with saline-injected group; # P<0.01, compared with LPS-injected group.

Two-way ANOVA analysis revealed statistical difference in the main effect of LPS (contact number: F = 0.417, P<0.5; contact time: F = 2.863, P<0.5), the main effect of treatment(contact number: F = 5.342, P<0.01; contact time: F = 6.144, P<0.01), and the interaction of the two factors (contact number: F = 3.960, P<0.05; contact time: F = 7.240, P<0.001). One ANOVA analysis revealed a significant effect among the eight groups in contact number [F(7,56) = 4.046; P<0.005] and contact time [F(7,56) = 6.145; P<0.001]. As shown in Figure. 2, post hoc analysis revealed that animals injected with LPS showed fewer contacts (P<0.001; Figure 3A) and less contact time (P<0.001; Figure 3B) compared with saline-injected group. These reductions in contact number and contact time in LPS-injected rats were rescued by the treatment of minocycline (P<0.001; Figure 3A and P<0.001; Figure 3B), risperidone (P<0.001; Figure 3A and P<0.001; Figure 3B) and both of them (P<0.001; Figure 3A and P<0.001; Figure 3B) as compared with LPS-injected rats. The data showed that the neonatal intrahippocampal LPS injection induces impaired social interaction in rats, and minocycline and risperidone are able to rescue this defective social interaction.

#### Novel object recognition test

**Figure 4:**
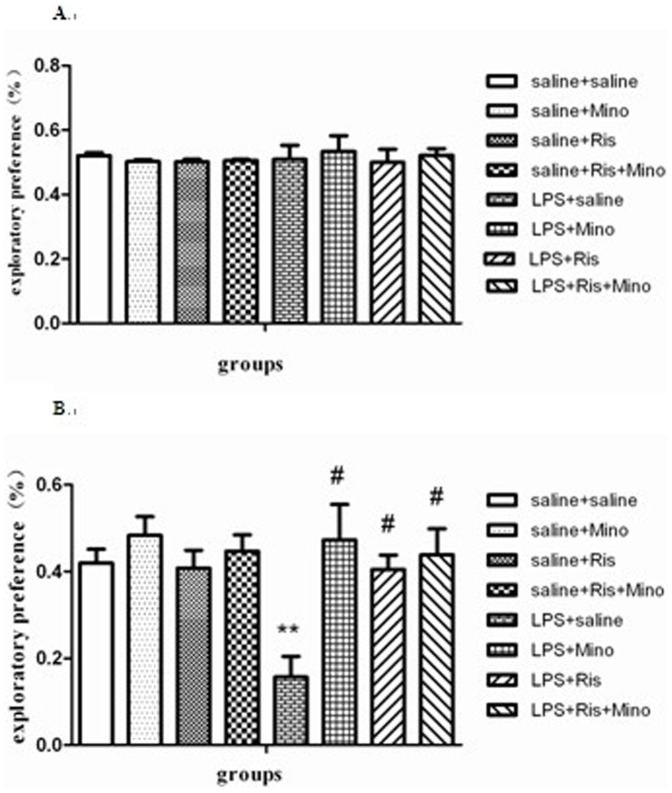
Effects of minocycline, risperidone and combination of these two on LPS-induced non-spatial memory deficits in rats (the training session as shown in Figure 4A and the retention session as shown in Figure 4B). The data is shown as means ± SEM. n = 8 for each group. ** P<0.01, compared with saline-injected group; # P<0.01, compared with LPS-injected group.

As shown in Figure 4A, in the training session, two-way ANOVA analysis revealed no statistical difference in the main effect of LPS (F = 0.170, P>0.5), the main effect of treatment(F = 0.135, P>0.5), or the interaction of the two factors (F = 0.219, P>0.5). In the retention session (Figure 4B), two-way ANOVA analysis revealed statistical difference in the main effect of LPS (F = 4.245, P<0.5), the main effect of treatment(F = 5.937, P<0.01), and the interaction of the two factors (F = 3.602, P<0.05). One-way ANOVA analysis revealed a significant effect among the eight groups [F(7,56) = 4.799; P<0.001]. Post hoc analysis revealed that the exploratory preference shown by the ratio of the amount of time spent in exploring the novel object over the total time spent exploring both objects in the LPS-injected group was significantly reduced compared with saline-injected group (P<0.001; Figure 4B). Interestingly, this defective non-spatial memory were rescued by the administration of minocycline (P<0.001; Figure 4B), risperidone (P<0.01; Figure 4B), and both of them (P<0.001; Figure 4B). Thus, minocycline and risperidone are capable of rescuing the non-spatial memory deficits.

#### PPI test

**Figure 5:**
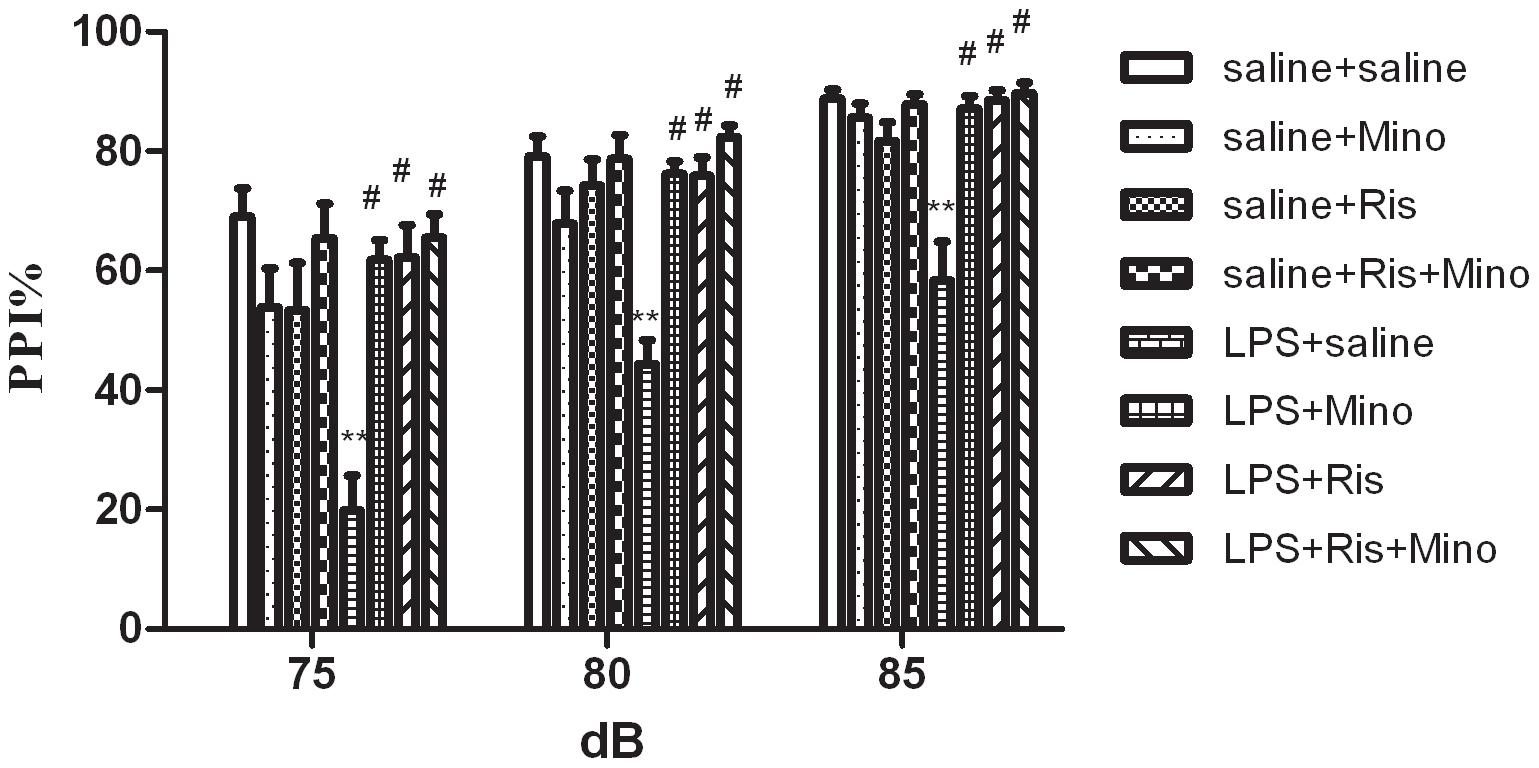
Effects of minocycline,risperidone, minocycline combination with risperidone on LPS-induced PPI deficits in rats. The data is shown as means ± SEM. n = 8 for each group. ** P<0.01. compared with saline-injected group; # P<0.01,compared with LPS-injected group.

Figure 5 shows the effects of minocycline, risperidone and both of them on LPS-induced PPI deficits in rats. The main effects of group treatment [F(1,7) = 22.353, P<0.001] and prepulse intensities [F(1,7) = 92.538, P<0.001] were significant. Subsequent ANOVA analysis revealed significant differences (all P<0.001) in all dB groups (75, 80, 85 dB). A posteriori analysis indicated a significant difference (P<0.01) between saline- and LPS-injected rats. Furthermore, a posteriori analysis demonstrated that minocycline, risperidone and both of the two attenuated significantly (all P<0.001) PPI deficits in rats received the neonatal hippocampal injection of LPS. The data suggest that the neonatal intrahippocampal LPS injection affects adult sensorimotor gating performance, and minocycline and risperidone are able to rescue the defective sensorimotor gating.

### Effects of minocycline, risperidone and combination of both of them on LPS-induced microglial activation

**Figure 6:**
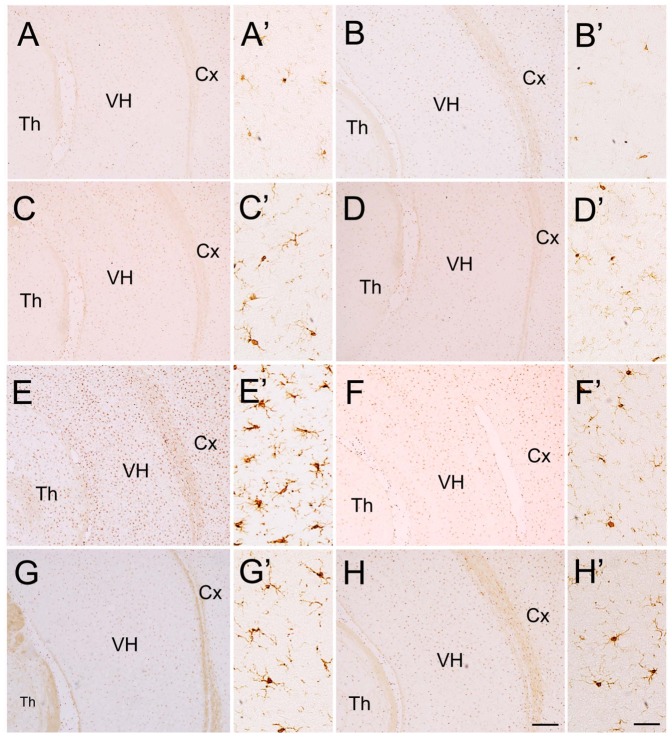
Iba1-immunopositive cells in VH, Cx and Th of saline- and LPS-injected rats. A small number of Iba1-immunopositive cells are present in VH, Cx and Th of rats received neonatal intrahippocampal injection of saline (A) and the saline-injected rats treated with minocycline (B), risperidone (C) or both of them (D). On the other hand, a large of number of Iba1-immunopositive cells are observed in VH, Cx and Th of the rats received neonatal intrahippocampal injection of LPS (E), but it is dramatically reduced after intragastric administration of minocycline (F), risperidone (G) or both of them (H). A'–H' are high magnification of the ventral hippocampus in A–H, respectively. Cx, cerebral cortex; Th, thalamus; VH, ventral hippocampus. Scale bars  = 200 μm in H (applied from A–G) and 20 μm in H' (applied for A'–G').

**Figure 7:**
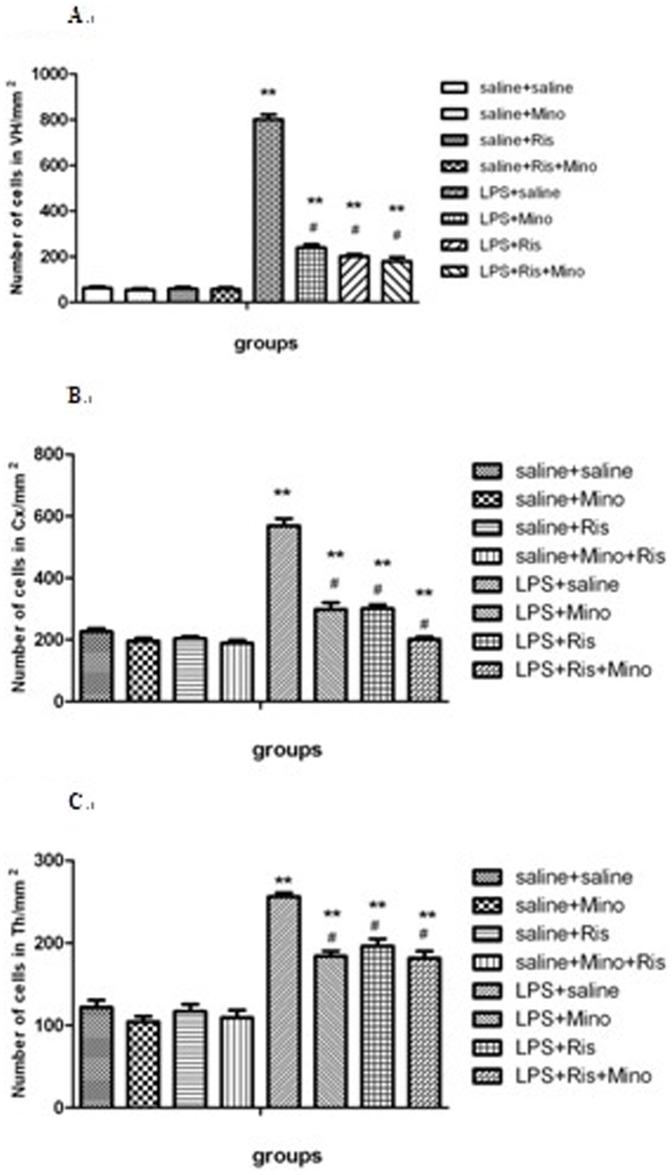
Comparison of number of Iba1-immunopositive cells in VH (A), Cx (B) and Th (C) among different groups. The data is shown as means ± SEM. n = 4 for each group. ** P<0.01, compared with saline-injected group; # P<0.01, compared with LPS-injected group.

In the brain of saline-injected rats, only a few Iba1-immunopositive cells were observed (Figure 6A). In the brain of LPS-injected rats, however, Iba1-immunopositive cells were dramatically increased in broad brain areas, including the cerebral cortex, thalamus and hippocampus (Figure 6E). Intragastric application of minocycline, risperidone or both of them had no effect on Iba1- immunoreactivity in the saline-injected rats (Figure 6B–D), but it markedly reduced number of Iba1-immunopositive cells in the LPS-injected rats (Figure 6F–H). We counted number of Iba1-immunopositive cells in the VH, Cx, and Th for statistical comparison. Two-way ANOVA analysis revealed statistical difference in the main effect of LPS [(VH: F = 1.121, P<0.001; Cx: F = 287.860, P<0.001); Th: F = 1.220, P<0.001)]; in the main effect of treatment[(VH: F = 287.810.121, P<0.001; Cx: F = 1.243, P<0.001); Th: F = 287.860.220, P<0.001)]; and the interaction of the two factors[(VH: F = 278.836, P<0.001; Cx: F = 278.836, P<0.001); Th: F = 278.836, P<0.001)]. Next, One ANOVA analysis revealed a significant effect among the eight groups in VH [F(7,24) = 402.989; P<0.001], Cx [F(7,24) = 85.76; P<0.001], Th [F(7,24) = 49.349; P<0.001]. As shown in Figure 7A–C, post hoc analysis revealed that number of Iba1-immunopositive cells in these brain regions of LPS-injected group was significantly increased compared with that in saline-injected group (all P<0.001). The increases were all significantly attenuated by the administration of minocycline (all P<0.001; Figure 7A–C), risperidone (all P<0.001; Figure 7A–C), and both of them (all P<0.001; Figure 7A-C) although it was still higher than that in saline-injected rats (all P<0.001; Figure 7A–C). Thus, the data indicated that microgia activation is widely present in the brain of LPS-injected rats, and minocycline and risperidone are able to attenuate the microglia activation.

## Discussion

The major findings of the present study are that minocycline and risperidone are able to rescue the behavioral deficits induced by the neonatal intrahippcampal LPS injection. In addition, microglia activation is present in the brain of LPS-injected rats, and it is largely suppressed by intragastric application of minocycline and risperidone. To our knowledge, this is the first report demonstrating that minocycline and risperidone are able to rescue the schizophrenia-like behavior as well as microglia activation induced by the neonatal intrahippocampal LPS.

Neonatal ventral hippocampal lesion is one of the most extensively used models for studying schizophrenia-like behaviors in animals [18]. A major drawback of this model, however, is the presence of the lesion, which is not typically observed in patients with schizophrenia. To avoid this, the intrahippocampal immune challenge model has been employed to induce schizophrenia-like behaviors in rats [10], and in this study we employed this new model to explore possible contribution of chronic neuroinflammation in the development of schizophrenia.

In animal models of schizophrenia, hyperlocomotion is thought to reflect a certain aspects of the positive symptoms [19] and deficits in social interaction are thought to correspond to some aspects of the negative symptoms of schizophrenia [20]. The PPI deficit that is observed in patients with schizophrenia correlates clinically to symptoms such as thought disorder and distractibility [20]. In addition, novel object recognition test is used to examine visual memory, and deficits in this test may show the reduction of motivation for a novel object and concentration ability as well as withdrawal symptoms; those might be related to cognitive deficits in schizophrenia [12]. Our data confirmed the previous findings that the neonatal intrahippocampal LPS injection serves an animal model in the examination of schizophrenia-like behaviors in rats. Except the hyperlocomotion, the other three typical behavior deficits induced by the intrahippocampal LPS injection were rescued by administration of minocycline or risperidone.

Microglial cells are the resident macrophage in the brain and the major players in innate immunity in the CNS. They are the primary reservoirs of pro-inflammatory cytokines such as IL-6, TNF-α and IFN-γ and act as antigen presenting cells in the CNS [1]. Recent studies have suggested the critical role of microglia in pathophysiology of schizophrenia. Two postmortem studies found activated microglia in the brains of patients of schizophrenia especially in the frontotemporal regions [5], [21], and PET analyses showed an increased number of microglial cells in patients with schizophrenia [6], [22]. In addition, it has been reported that the neonatal intrahippocampal injection of LPS results in persistent elevation in cytokines in several brain regions [10], and risperidone inhibits the release of the pro-inflammatory cytokines (e.g. IL-1 β, IL-6, and TNF-α) by activated microglia *in vitro* [23]. In this study, we found that in intrahippocampal LPS-injected rats a drastic activation of microglia is widely present in the brain, and this activation is attenuated and the schizophrenia-like behaviors are rescued by risperidone or minocycline. We speculate that LPS injection in the ventral hippocampus induces significantly increased microglia activation in the ventral hippocampus first and the activated microglia could produce cytokins and free radicals, and these factors, in turn, affect Iba1 expression in the brain regions outside hippocampus. On the basis of the present and previous findings, it is very likely that the microglia activation and subsequent excessive release of pro-inflammatory cytokines contribute to the development of schizophrenia. However, although it is well accepted that LPS treatment leads to immune responses including microglia activation, it may also result in other unknown effects that contributes to the behavioral alterations in our animal model. In addition, we showed the decrease of microglia in LPS-treated rats after the application of risperidone and minocycline, but there is no direct evidence to establish a causal link between microglia activation and rescued schizophrenia-like behavior. Further studies are needed to explore this interesting question.

It was reported that [24] minocycline significantly attenuated methamphetamine-induced hyperlocomotion and N-methyl-D-aspartic acid (NMDA) receptor antagonist dizocilpine-induced PPI deficits in mice [11]. In addition, clinical studies showed that adjunctive therapy of minocycline to antipsychotics is beneficial for the treatment of the patients with schizophrenia [7], [25]. Our data revealed that minocycline could alleviate the schizophrenia-like behavioral changes in intrahippocampal LPS-injected rats. These data suggest that anti-inflammatory treatment should be encouraged in the therapy of schizophrenia.

In this study, we found that minocycline and risperidone were individually able to rescue the behavioral deficits and inhibit the microglia activation in intrahippocampal LPS-injected rats, which suggests that inhibition of microglial activation may be one of mechanisms of the antipsychotic effect of minocycline and risperidone. On the other hand, previous studies have reported that risperidone also suppresses proinflammatory cytokines and upregulates anti-inflammatory cytokines [26].

Moreover, minocycline also has the property of agnostic action on NMDA receptors [27]. Previous studies have reported that microglia cells also express certain receptors for neurotransmitters/modulaters, such as ATP, adenosine, glutamate, GABA, acetylcholine, dopamine, serotonin, and adrenaline [28]. Particularly, some neurotransmitters play an important role in controlling neuron-microglia interactions during inflammatory processes in disease progression [28]. It has been proposed that modulating microglia cells has the potential for treating schizophrenia [1]. Thus, in addition to the reduction of microglia activation, other mechanisms such as proinflammatory or neurotransmitter regulations may also be involved. Kato et al found that risperidone [23] significantly inhibited the production of NO and proinflammatory cytokines by activated microglia in vitro and aripiprazole [29] may have psychotropic effects by reducing the microglial oxidative reactions and following neuronal reactions, which suggest that there are other antipsychotics except risperidone have the inhibition effect on microglia activation. It should be noted that application of both of them together did not show an additive effect. One of possible explanations is that the two drugs may function through the same pathway (e.g. chronic neuroinflammatory pathway), and thus blocking or suppressing any component of the pathway leads to the same effects. It is also likely that they act on different pathways, and their effects on rescuing behavioral deficits and suppressing microglia activation are at the same level or reach the maximum level (the ceiling effect). On the other hand, to clearly identify the effects of risperidone and minocycline on the behavioral deficits and microglial activation in LPS-treated rats, dose response should be examined in future studies.

## Conclusion

In summary, the present results confirmed the previous findings that neonatal intrahippocampal injection of LPS induces schizophrenia-like behavioral deficits, and further support the idea that this is a reliable schizophrenia-like animal model.

Moreover, minocycline and risperidone attenuate these behavioral changes and inhibit the microglia activation in the brain, suggesting that inhibition of microglia activation may be one of mechanisms of the antipsychotic effect of minocycline and risperidone.
